# Distinct Temporal and Anatomical Distributions of Amyloid-β and Tau Abnormalities following Controlled Cortical Impact in Transgenic Mice

**DOI:** 10.1371/journal.pone.0025475

**Published:** 2011-09-29

**Authors:** Hien T. Tran, Laura Sanchez, Thomas J. Esparza, David L. Brody

**Affiliations:** 1 Department of Neurology, Washington University in St. Louis, St. Louis, Missouri, United States of America; 2 Hope Center for Neurological Disorders, Washington University in St. Louis, St. Louis, Missouri, United States of America; Boston University School of Medicine, United States of America

## Abstract

Traumatic brain injury (TBI) is a major environmental risk factor for Alzheimer's disease. Intracellular accumulations of amyloid-β and tau proteins have been observed within hours following severe TBI in humans. Similar abnormalities have been recapitulated in young 3xTg-AD mice subjected to the controlled cortical impact model (CCI) of TBI and sacrificed at 24 h and 7 days post injury. This study investigated the temporal and anatomical distributions of amyloid-β and tau abnormalities from 1 h to 24 h post injury in the same model. Intra-axonal amyloid-β accumulation in the fimbria was detected as early as 1 hour and increased monotonically over 24 hours following injury. Tau immunoreactivity in the fimbria and amygdala had a biphasic time course with peaks at 1 hour and 24 hours, while tau immunoreactivity in the contralateral CA1 rose in a delayed fashion starting at 12 hours after injury. Furthermore, rapid intra-axonal amyloid-β accumulation was similarly observed post controlled cortical injury in APP/PS1 mice, another transgenic Alzheimer's disease mouse model. Acute increases in total and phospho-tau immunoreactivity were also evident in single transgenic Tau_P301L_ mice subjected to controlled cortical injury. These data provide further evidence for the causal effects of moderately severe contusional TBI on acceleration of acute Alzheimer-related abnormalities and the independent relationship between amyloid-β and tau in this setting.

## Introduction

Moderate to severe traumatic brain injury (TBI) can accelerate cognitive decline and increases the risk of dementia of the Alzheimer's type [1], [2], [3], [4], [5]. Alzheimer's disease (AD) is characterized by several pathological hallmarks, including tau-containing neurofibrillary tangles and neuritic plaques composed of the amyloid-β (Aβ) peptides [6]. There has been robust evidence linking TBI to AD-related pathologies. Intracellular accumulation of Aβ, extracellular deposition of diffuse Aβ plaques, and aggregation of tau have been observed in humans, sometimes within hours post severe injury [7], [8], [9], [10], [11], [12], [13]. Therefore, TBI is hypothesized to be causally related to acceleration of AD-related pathologies. Rotational head injury in pigs [14] and our recent findings in young 3xTg-AD mice subjected to CCI support this hypothesis [15]. Specifically, we found intra-axonal Aβ accumulation and accelerated tau pathology in these mice at 1 day and 7 days post TBI. There has been some controversy about whether the intracellular immunoreactivity using certain antibodies represents Aβ vs. APP [16]. Our immunostaining using several antibodies including 3D6 established that this post-injury axonal immunoreactivity was specific for Aβ [15], as 3D6 does not recognize APP [17]. The questions of whether Aβ and tau pathologies are altered within hours post TBI and whether the findings in 3xTg-AD mice can be generalized remained to be investigated. In the current study, we show that Aβ accumulation is observed as early as 1 hour post injury in 3xTg-AD mice, and the temporal pattern of Aβ accumulation is distinct from those of tau abnormalities. Additionally, we demonstrate that CCI also causes acute Aβ accumulation in young APP/PS1 mice [18], which harbor a different PS1 mutation from 3xTg-AD mice, and acutely accelerates tau pathology in Tau_P301L_ transgenic mice [19]. Overall, our CCI model represents a useful tool for future investigation into the link between TBI and AD.

## Results

### Acute axonal Aβ pathology post CCI in 3xTg-AD mice

Axonal Aβ pathology is a characteristic feature of human traumatic axonal injury [9], [13], [20]. To model this pathology, we employed CCI TBI on young 3xTg-AD mice, which express mutant forms of human amyloid precursor protein (APP), presenilin 1 (PS1) and tau [21], [22]. By staining the brains of injured and age-matched, uninjured 3xTg-AD mice with several different antibodies specific for Aβ, we have previously shown that this injury paradigm caused intra-axonal Aβ accumulation at 24 h post TBI [15].

**Figure 1:**
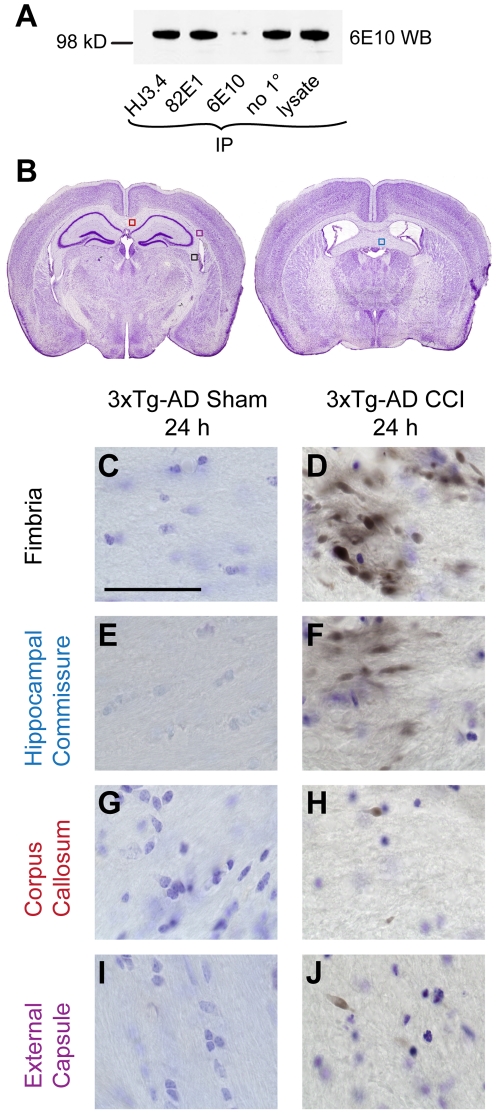
Controlled cortical impact (CCI) causes intra-axonal Aβ accumulation in young 3xTg-AD mice at 24 hours. **A**. Immunoprecipitation (IP) and Western blot (WB) showed that HJ3.4 antibody, similar to 82E1 antibody, did not recognize APP, while, 6E10 antibody recognized APP. **B**. Representative Cresyl violet stained sections showing white matter regions (boxes) with positive axonal Aβ pathology following TBI in 3xTg-AD mice (Modified from [46]). **C–J**. HJ3.4 staining in uninjured (sham) and injured (CCI) 3xTg-AD mice, counterstained with Cresyl violet. Intra-axonal Aβ accumulation was observed in the ipsilateral fimbria (**C–D**, black box in **B**), ipsilateral hippocampal commissure (**E–F**, blue box in **B**), ipsilateral corpus callosum (**G–H**, red box in **B**) and ipsilateral external capsule (**I–J**, purple box in **B**) of injured 3xTg-AD mice. Scale bar in **C**: 50 µm. Most prominent Aβ staining was observed in the ipsilateral fimbria/fornix of injured mice. Aβ staining has beads-on-a-string and varicose morphologies, consistent with morphologies of injured axons.

We analyzed Aβ axonal pathology with HJ3.4 antibody against Aβ_1–13_ in these studies. To demonstrate that HJ3.4 does not recognize APP, we performed immunoprecipitation followed by a Western blot analysis. Identical aliquots (100 µg) from brain lysates of a 9 month-old 3xTg-AD mouse were immunoprecipitated with monoclonal HJ3.4, 82E1, 6E10 antibodies, or no primary antibody control. Monoclonal 82E1 has been previously shown to be specific for Aβ [16], [23], while monoclonal 6E10 antibody can recognize both Aβ and APP [16]. The resultant immunodepleted supernatants were subjected to Western blotting with 6E10 antibody. Our data demonstrated that HJ3.4 antibody, similar to 82E1 antibody, does not immunoprecipitate APP (**Figure 1A**).

When we stained the brains of injured and sham 3xTg-AD mice which were sacrificed at 24 hours post injury with HJ3.4 antibody, we observed that the fimbria/fornix, a white matter region vulnerable to axonal injury, exhibited the most prominent axonal Aβ pathology (**Figure 1B, D**). This was consistent with our previous findings using other anti-Aβ antibodies [15]. Less extensive but still clearly abnormal Aβ accumulation was observed in the ipsilateral hippocampal commissure (**Figure 1F**), corpus callosum (**Figure 1H**), and external capsule (**Figure 1J**) of injured 3xTg-AD mice. Aβ was not immunohistochemically detected in the corresponding white matter regions of age-matched, uninjured 3xTg-AD mice (**Figure 1 C, E, G, I**). No Aβ staining was observed in the ipsilateral CA1 of injured 3xTg-AD mice (data not shown).

**Figure 2:**
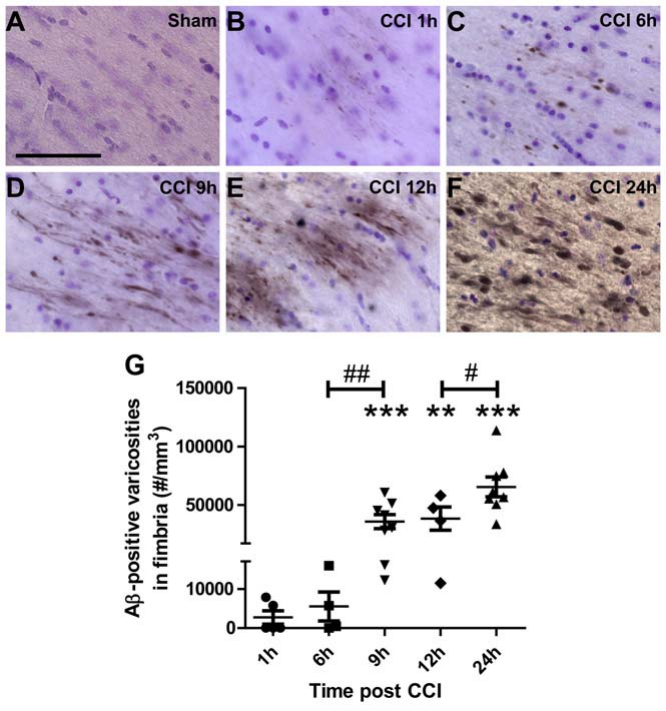
Intra-axonal Aβ accumulation monotonically increases from 1 to 24 hours post CCI in 3xTg-AD mice. **A**. Aβ staining with biotinylated HJ3.4 antibody (against Aβ_1–13_) in the ipsilateral fimbria/fornix of a sham 3xTg-AD mouse. Sections were counterstained with Cresyl violet. Scale bar: 50 µm. **B–F**. Aβ staining in the ipsilateral fimbria/fornix of an injured 3xTg-AD mouse at 1 h (**B**), 6 h (**C**), 9 h (**D**), 12 h (**E**) and 24 h (**F**) after CCI. **G**. Stereological quantification of total numbers of Aβ-positive axonal varicosities as a function of time after injury in 3xTg-AD mice. N = 4–8 mice per group per time point. Bars represent mean ± SEM. One-way ANOVA with Newman-Keuls post tests, # p<0.05, ## p<0.01: significant increase from injured mice from previous time point. ** p<0.01, *** p<0.0001: significant increase from sham mice at same time point.

Since Aβ accumulation has been detected as early as 2 h post severe TBI in humans [7], we tested the hypothesis that TBI causes very early axonal Aβ accumulation in 3xTg-AD mice by sacrificing independent groups of mice at 1, 6, 9, 12, and 24 h post injury. We found Aβ in injured axons at all time points following injury (**Figure 2**). Morphologies of Aβ-positive axonal varicosities evolved from small swellings observed at 1 and 6 hours after injury (**Figure 2B–C**) to larger spheroids, bulbs, and beaded varicose fibers at the later times (9, 12, and 24 h post TBI, **Figure 2D–F**). Stereological quantification revealed moderate numbers of injured axons with Aβ accumulation in some but not all mice at the earliest time points examined (1 h and 6 h after injury, **Figure 2G**). However, substantially greater numbers of Aβ-immunoreactive axonal varicosities were present at later time points, and all mice sacrificed between 9 and 24 hours had this pathology (9, 12, and 24 h, **Figure 2G**). The increase in Aβ-positive axonal varicosities between 6 and 9 hours after TBI was statistically significant, as was the increase between 12 and 24 hours (**Figure 2G**, p<0.05).

In summary, CCI TBI consistently accelerated Aβ axonal accumulation in young 3xTg-AD mice. Aβ accumulation appeared as early as 1 h post TBI, and continued to rise through 24 h.

### Aβ accumulation in APP/PS1 mice

**Figure 3:**
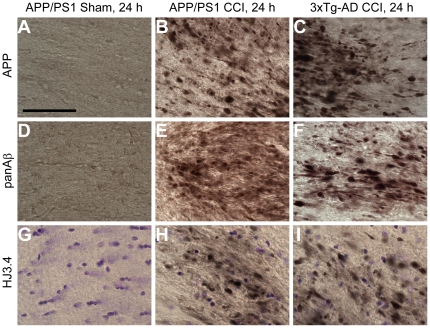
Aβ accumulates in fimbria/fornix axons of 2 month-old APP/PS1 mice at 24 hours post CCI. **A–C**. APP staining in the ipsilateral fimbria/fornix of a sham APP/PS1 mouse (**A**), an injured APP/PS1 mouse (**B**), and an injured 3xTg-AD mouse (**C**). Scale bar: 50 µm. Similar extent of axonal injury as detected by APP staining was seen in injured APP/PS1 and 3xTg-AD mice. **D–F**. Aβ staining with panAβ antibody. **G–I**. Aβ staining with HJ3.4 antibody. Histological and stereological quantification showed similar extent of Aβ accumulation in injured APP/PS1 and 3xTg-AD mice: 47,257±11,763 HJ3.4-positive varicosities per cubic mm in APP/PS1 (n = 5) vs. 65,437±8,458 in 3xTg-AD mice (n = 8), Student's t-test, p = 0.23.

To test whether the findings of acute Aβ accumulation post TBI in 3xTg-AD mice can be generalized to another mouse model, we subjected a different transgenic line, APP/PS1 mice to CCI of similar injury severity. These mice overexpress the Swedish (K670M/N671L) mutation of the human APP gene and the human PS1 gene with exon 9 deleted [18]. They were injured at 2 months of age; extensive extracellular Aβ pathology normally develops by approximately 6 months of age in this line. They were sacrificed at 24 h post TBI; their brains were stained for APP to assess the extent of axonal injury, and for Aβ using two different antibodies against Aβ: panAβ polyclonal and HJ3.4 monoclonal antibodies. TBI resulted in comparable degree of axonal injury in pericontusional white matter in both APP/PS1 and 3xTg-AD mice, as evidenced by similar patterns of APP staining (**Figure 3B–C**). Stereological quantification of APP-positive axonal varicosities corroborated the qualitative observation (3xTg-AD: 295,579±36,388 APP-positive axonal varicosities per cubic mm, n = 8 vs. APP/PS1: 272,212±43,249, n = 5, p = 0.69). Likewise, the pattern of Aβ accumulation detected by panAβ and HJ3.4 antibodies appeared similar in injured 3xTg-AD and APP/PS1 mice (**Figure 3E–F, H–I**). Quantification also confirmed this histological finding (3xTg-AD: 65,437±8,458 HJ3.4-positive varicosities vs. APP/PS1: 47,257±11,763, p = 0.23). Uninjured APP/PS1 mice at 2 months of age had neither APP nor Aβ accumulation in the ipsilateral fimbria/fornix (**Figure 3A, D, G**). These data suggest post-traumatic Aβ accumulation in 3xTg-AD mice is not unique to the genetic constructs carried by these mice.

### Anatomical and temporal patterns of tau accumulation post CCI in 3xTg-AD mice

**Figure 4:**
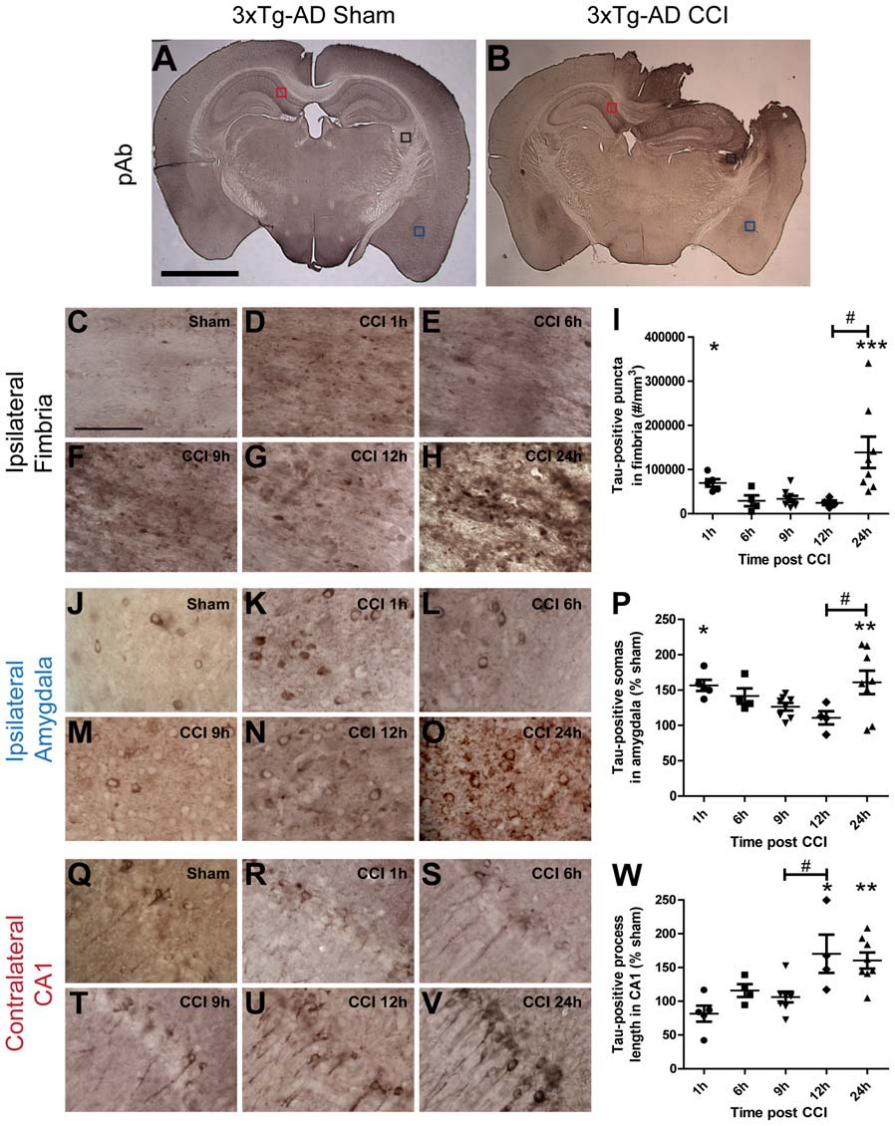
Time course of CCI-induced tau pathology is distinct in several brain structures of 3xTg-AD mice. Tau staining was with polyclonal total tau antibody. **A–B**. Tau staining in a sham (**A**) and an injured (**B**) 3xTg-AD mouse. Three regions with accelerated tau pathology compared to sham at 24 h post TBI were the ipsilateral fimbria/fornix (black box), ipsilateral amygdala (blue box), and contralateral CA1 (red box). Scale bar: 2 mm. **C–H**. Higher magnification of punctate tau staining in the ipsilateral fimbria (black box in **A–B**) of a sham (**C**) and injured 3xTg-AD mice at 1 h (**D**), 6 h (**E**), 9 h (**F**),12 h (**G**), and 24 h (**H**) following CCI. Scale bar in C: 50 µm. **I**. Stereological quantification of numbers of tau-positive puncta per cubic millimeter in ipsilateral fimbria as a function of time post injury. **J–O**. Perinuclear, cytoplasmic tau staining in somata of the ipsilateral amygdala (blue box in **A–B**). **P**. Stereological quantification of numbers of tau-positive somata per cubic millimeter in the ipsilateral amygdala as a function of time post injury. **Q–V**. Tau staining in processes of the contralateral CA1 (red box in **A–B**). **W**. Stereologcial quantification of tau-positive processes of CA1 pyramidal neurons per cubic millimeter as a function of time post injury. N = 4–8 mice per group per time point. Bars are mean ± SEM in fimbria, and percent of sham ± SEM for amygdala and CA1 region. One-way ANOVA with Newman-Keuls post tests, * p<0.05, ** p<0.01, *** p<0.001: significant increase from sham mice at same time point. # p<0.05: significant increase from injured mice at previous time point.

We have previously reported that CCI resulted in tau accumulation at 24 h in several brain regions of injured 3xTg-AD mice [15]. These were the ipsilateral fimbria/fornix, ipsilateral amygdala, and contralateral (but not ipsilateral) CA1 (**Figure 4A–B**). Total tau accumulated in puncta in the ipsilateral fimbria/fornix. Perinuclear tau staining was observed in neurons of the ipsilateral amygdala, while tau staining mostly localized to neuronal processes of the contralateral CA1.

Here, we investigated the temporal patterns of tau accumulation in these regions using the same mice used to assess the time course of Aβ described above. We quantitatively characterized the time course of tau immunoreactive changes using stereological methods (**Figure 4I, P, W**). In the ipsilateral fimbria, there were significantly elevated numbers of tau-positive puncta at 1 h and 24 h, but not at 6 h–12 h following injury (**Figure 4C–H**). In sham mice, there were 3,420±919, whereas at 1 h post injury, there were 69,641±8,496 (p<0.05) and at 24 h there were 138,887±35,543 (p<0.0001) tau-stained puncta per cubic millimeter of fimbria (**Figure 4I**).

Tau immunoreactivity in cell bodies of the ipsilateral amygdala exhibited a similar biphasic time course: the numbers of immunoreactive cell bodies were increased at 1 h following injury (**Figure 4K**), came back to sham levels from 6 h to12 h (**Figure 4L–N**), and rose again at 24 h (**Figure 4O**). Since there was substantial tau immunoreactivity in sham 3xTg-AD mice in this region (**Figure 4J**), stereological quantification of numbers of tau-positive somata was expressed as percent of sham. While numbers of tau-positive cell bodies from 6 h to 12 h after injury were similar to sham, significantly more were apparent at 1 h and 24 h in ipsilateral amygdala after injury (**Figure 4P**, p<0.05).

Interestingly, the temporal profile of tau-positive processes in the contralateral hippocampal CA1 region followed a different pattern, with a delayed monophasic rise. Specifically, the extent of tau immunoreactivity in contralateral CA1 in uninjured 3xTg-AD mice and injured mice sacrificed from 1 h to 9 h following injury appeared similar (**Figure 4Q–T**). From 12 h after TBI, however, tau immunoreactivity in this region increased (**Figure 4U–V**). Stereological quantification of total length of tau-positive process using the spherical probes (also known as ‘spaceballs’) method indicated a significant increase from sham starting at 12 h following injury (**Figure 4W**, p<0.05); this measure remained elevated at 24 h (**Figure 4V, W**).

Thus, CCI increased tau immunoreactivity in a multifocal fashion in the brains of 3xTg-AD mice. A two-phase increase in tau immunoreactivity was observed at 1 hour and 24 hours after TBI in the ipsilateral fimbria and ipsilateral amygdala, while only a single phase was observed at 12–24 hours in the contralateral hippocampal CA1 region. Notably, the anatomical and temporal distribution of TBI-related changes in tau immunoreactivity was distinct from those of post-injury Aβ accumulation.

### Increased tau immunoreactivity in Tau_P301L_ mice post CCI

**Figure 5:**
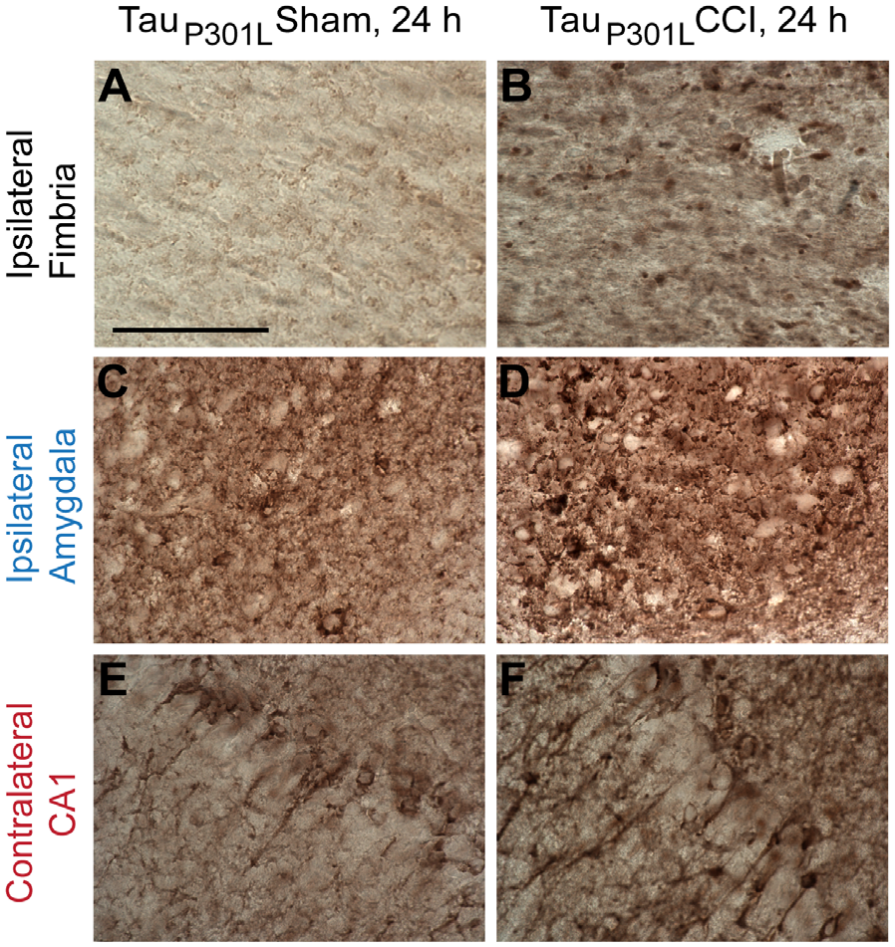
CCI causes acute axonal tau accumulation and increases somatic tau staining in Tau_P301L_ mice. Polyclonal antibody against total human tau was used for immunohistochemistry. **A–B**. Punctate tau staining in the ipsilateral fimbria of injured but not sham Tau_P301L_ mice. Scale bar in A: 50 µm. **C–D**. Increased tau immunoreactivity in the cell bodies of the ipsilateral amygdala of injured compared to sham Tau_P301L_ mice. **E–F**. Increased tau immunoreactivity in processes of the contralateral CA1 of injured compared to sham Tau_P301L_ mice.

To provide further evidence for the independent relationship between Aβ and tau in the setting of TBI, we performed CCI on transgenic mice expressing only human tau mutant gene, Tau_P301L_ [19]. Expression of the transgene in these mice was under transcriptional control of the Thy1.2 promoter, the same promoter which drives transgenes expression in 3xTg-AD mice. Tau pathology was investigated at 24 h post TBI in 6 month old Tau_P301L_ mice by immunohistochemistry with an antibody against total human tau. We found that CCI also caused acute tau accumulations with punctate morphologies in the ipsilateral fimbria/fornix of injured Tau_P301L_ mice (**Figure 5A–B**). Quantification indicated there were substantial numbers of tau-positive puncta in the ipsilateral fimbria/fornix of injured Tau_P30L_ mice; approximately half as many as in injured 3xTg-AD mice (Tau_P301L_: 63,180±9,636 tau-positive puncta per cubic mm of fimbria, n = 6, vs. 3xTg-AD mice: 138,887±35,543, n = 8, p = 0.1). This result is not surprising, as the 3xTg-AD mice were homozygous for human mutant tau whereas the Tau_P301L_ mice were heterozygous. Furthermore, total tau staining in the ipsilateral amygdala and contralateral CA1 of injured Tau_P301L_ was increased relative to sham Tau_P301L_ mice, similar to the effects in injured 3xTg-AD mice (**Figure 5C–F** vs. **Figure 4O, V**).

**Figure 6:**
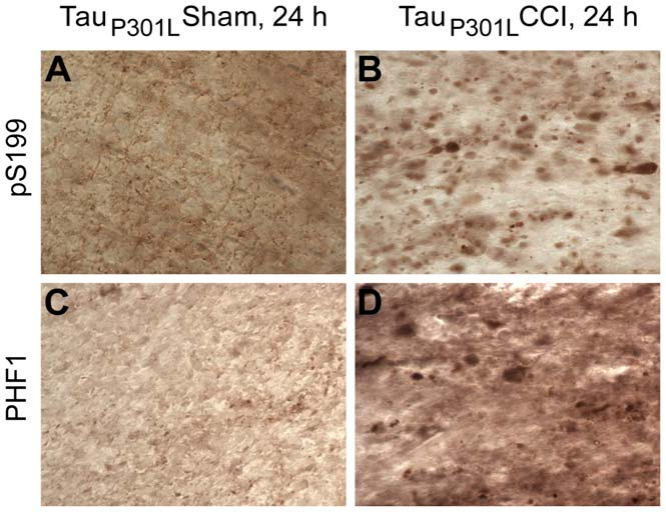
CCI affects tau phosphorylation in the ipsilateral fimbria/fornix of Tau_P301L_ mice at 24 hours. **A–B**. Phospho-tau staining using pS199 antibody against tau phosphorylated at S199. Scale bar in A: 50 µm. **C–D**. Phospho-tau staining using PHF1 antibody against tau phosphorylated at S396 and S404. Both phospho-tau antibodies detect punctate axonal tau accumulations in the ipsilateral fimbria of injured but not sham Tau_P301L_ mice.

Since CCI acutely affects tau phosphorylation in 3xTg-AD mice at several sites, specifically at Serine 199, Serine 396 and Serine 404, as detected with phospho-specific tau antibodies pS199 and PHF1, respectively [15], we tested whether CCI increased tau phosphorylation in injured Tau_P301L_ mice by staining with these antibodies. Abnormal, punctate phospho-tau staining was observed in the ipsilateral fimbria/fornix of injured but not uninjured Tau_P301L_ mice (**Figure 6A–D**). Similar results were found in all 6 injured Tau_P301L_ mice.

In summary, controlled cortical impact TBI consistently increased tau pathology in both young 3xTg-AD mice and Tau_P301L_ mice. The anatomical and temporal pattern of tau pathology was distinct from that of Aβ.

## Discussion

The current study shows that CCI TBI can cause rapid Aβ accumulation in injured axons of young 3xTg-AD mice. This intra-axonal Aβ was detectable at 1 hour post injury, and continued to rise monotonically through 24 hours. Several brain regions of injured 3xTg-AD mice also exhibited increased tau immunoreactivity, but the time course was different across regions. In particular, puntate tau staining the ipsilateral fimbria and perinuclear tau staining in the amygdala had a biphasic response with peaks at 1 hour and 24 hours post TBI. Instead, the numbers of tau-positive processes in the contralateral CA1 started to increase at 12 h post injury. However, total tau immunoreactivity in the ipsilateral CA1 of 3xTg-AD mice was not significantly affected by TBI. Neuronal damage to this region may have caused release of tau into the extracellular space, where it could not be detected by immunohistochemistry. Importantly, the finding of post-traumatic Aβ accumulation in 3xTg-AD mice was recapitulated in a different transgenic mouse model of Alzheimer's disease, APP/PS1. Similarly,accelerated tau pathology in 3xTg-AD mice was also observed in transgenic mice carrying only Tau_P301L_ mutation at 24 hours following TBI.

We have previously presented evidence that CCI can independently alter Aβ and tau abnormalities in 3xTg-AD mice [15]. Specifically, systemic inhibition of γ-secretase activity, an enzyme required for Aβ generation from its precursor, APP, successfully blocked post-traumatic Aβ accumulation in injured mice. However, tau pathology was unaffected following blockade of Aβ generation and accumulation. In the present study, we found distinct anatomical and temporal patterns of Aβ and total tau abnormalities throughout 24 hours post TBI in 3xTg-AD mice. Furthermore, we found abnormal total and phospho-tau accumulation in injured axons, and increased somatic tau staining in single-transgenic Tau_P301L_ mice subjected to TBI. Although the temporal distribution of phospho-tau following acute TBI in 3xTg-AD mice remains to be investigated, findings in this study add additional support to the hypothesis that Aβ and tau pathologies are independent in the setting of TBI. As such, future studies will be required to investigate the mechanisms underlying TBI-induced tau hyperphosphorylation.

APP, the precursor protein of Aβ, has been found to accumulate in injured axons within 30 minutes following central nervous system injury [24]. Axonal APP accumulation has in turn been hypothesized to serve as substrate for intra-axonal Aβ generation [20], [25]. Thus, our finding that intra-axonal Aβ was detected starting at 1 hour post TBI in 3xTg-AD mice is in line with the reported time for the earliest APP accumulation following brain trauma.

PS1 mutations are thought to drive intracellular Aβ generation [26]. Additionally, transgenic mice which have both PS1 mutations and APP mutations exhibit accelerated Aβ pathology compared to those with only APP mutations [18], [27], [28], [29], [30], [31]. In the setting of TBI, these mutations also appear necessary for rapid intra-axonal Aβ accumulations. Acute Aβ accumulation in axons of injured 3xTg-AD and APP/PS1 mice in the present study and the lack of such pathology in previous experimental TBI models using wildtype and mutant APP mice without PS1 mutations support this observation [32].

Our mouse model recapitulates one aspect of post-traumatic Aβ pathology in human TBI: intra-axonal Aβ accumulation. Neither our model nor other small animal experimental TBI models of which we are aware result in acute extracellular plaques. Interestingly, recent findings suggest that intracellular Aβ accumulation is an early event in Alzheimer's disease pathogenesis, preceding plaque formation [33], [34], [35], [36]. Indeed, animal and cellular Alzheimer models have shown that the accumulation of intracellular Aβ species are neurotoxic and may be linked to synaptic dysfunction, cell loss, and memory impairment [26], [28], [37], [38], [39]. Thus, our TBI mouse model of intra-axonal Aβ accumulation may emerge as an interesting model to study the relationship between TBI and Alzheimer's disease.

The biphasic increase in tau immunoreactivity following TBI in ipsilateral fimbria and amygdala of 3xTg-AD mice is intriguing. Changes of tau immunoreactivity at 1 hour post TBI perhaps reflect an immediate response to mechanical injury. Increased tau immunoreactivity at 24 hours in this study, together with our previous finding of persistent tau abnormalities at 7 days, suggests initiation of secondary injury mechanisms induced by TBI.

However, our TBI mouse models have several limitations. First, we utilized transgenic mice with mutations implicated in familial dementia, while most humans with TBI are not genetically predisposed to developing such Aβ and tau pathologies. Nevertheless, these mutations seem to be required for post-traumatic human pathologies to be recapitulated in mice. Other genetic differences between humans and mice may be one of the underlying reasons. Second, the majority of brain injuries in human are mild and diffuse [40], while our TBI model produces a relatively severe, focal contusion with pericontusional axonal injury. Thus, to generalize our findings, other TBI paradigms such as fluid percussion injury and closed-skull impact, which result in more diffuse axonal injury, will be required. Lastly, the current study focuses only on the acute period post injury. Future studies will therefore be required to assess the long-term effects of intra-axonal buildup of Aβ and tau on neuronal survival, synaptic integrity, and behavioral outcomes following TBI in these mice.

In summary, our experimental TBI model using 3xTg-AD, APP/PS1, and Tau_P301L_ mice confirms that moderately severe CCI TBI can acutely accelerate intra-axonal Aβ and tau pathologies, and increase cytoplasmic tau accumulation. These models may provide useful tools to study therapeutic strategies to prevent adverse effects mediated by these pathologies following brain injury.

## Materials and Methods

### Animals

We used 6 month old homozygous 3xTg-AD mice, 2 month old heterozygous APP_swe_/PSEN1ΔE9 (APP/PS1) transgenic mice (line 85, Stock number 004462, The Jackson Laboratory), and 6 month old heterozygous Tau_P301L_ mice. 3xTg-AD mice have human PS1_M146V_ gene knocked in to the mouse allele, overexpress human APP Swedish gene and tau_P301L_ gene [22]. 3xTg-AD mice used for all experiments were derived from the original founders received from the LaFerla lab in 2007. There was no evidence of genetic drift over time. APP/PS1 mice overexpress human APP Swedish gene and human *PSEN1* with an exon 9 deletion [18]. Tau_P301L_ mice overexpress human tau gene with P301L mutation [19]. Mice were housed in standard cages in 12 h light, 12 h dark cycle and given food and water *ad. lib*. Mice of both sexes were randomly assigned to experimental groups. All experiments were approved by the animal studies committee at Washington University in St Louis, animal welfare assurance number A-3381-01.

### Controlled cortical impact experimental TBI

The experimental TBI methods used in this study were performed as described previously [41], [42]. Briefly, following craniotomy, experimental TBI was induced by impacting a 3.0 mm diameter metal tip onto the cortex (5 m/s, 100 ms dwell time). A 2.0 mm impact below the dura was chosen for all experiments, as this injury severity results in contusion in the ipsilateral cortex and substantial damage to the underlying hippocampus. Sham injured mice went through similar surgical procedures but were not injured. Mice were kept at 37°C throughout the procedure and allowed to recover on a warming pad to prevent hypothermia-induced hyperphosphorylation of tau [43].

### Antibodies

Monoclonal HJ3.4 antibody (against Aβ_1–13_) was a gift from Dr. David Holtzman [44]. Monoclonal 82E1 antibody, which recognizes free N-terminus of Aβ [16], [23], was purchased from IBL-America (Minneapolis, MN). Monoclonal 6E10 antibody, which recognizes both APP and Aβ [16], was from Covance (Princeton, NJ). Polyclonal APP, panAβ (against Aβ_15–30_), and phospho-tau at S199 antibodies were purchased from Invitrogen Corp. Polyclonal total tau antibody was from Pierce. Monoclonal PHF1 antibody (against tau phosphorylated at S396 and S404) was a gift from Dr. Peter Davies, Albert Einstein College of Medicine [45]. HJ3.4 was biotinylated using commercially available reagent from Pierce.

### Immunoprecipitation and Western Blot

To verify the specificity of HJ3.4 for Aβ over amyloid precursor protein (APP), an immunodepletion assay was performed on brain homogenate from a 9 month old 3xTg-AD mouse. Whole brain was removed after transcardial perfusion with PBS containing 0.3% heparin and immediately dounce homogenized in RIPA buffer (150 mM NaCl, 50 mM Tris-HCl, 1% Triton X-100, 0.10% SDS, 0.5% deoxycholic acid, 2.5 mM EDTA, pH 8.0) containing protease inhibitor cocktail (Roche) at a 10∶1 ratio (RIPA volume/tissue weight) using 25 strokes followed by brief sonication. The resulting homogenate was centrifuged for 20 minutes at 17,000×g at 4°C to remove insoluble protein. Total protein was determined using a standard BCA protein assay. Individual aliquots containing 100 µg of homogenate were immunodepleted using 10 µg of each antibody (HJ3.4, 82E1, 6E10). After overnight incubation, complexes were captured using 150 µg Protein-G Dynabeads® (#100.03D, Invitrogen). The resulting immunodepleted supernatants were assayed by Western blot, as described below, to determine affinity in solution for APP.

Samples for Western blot analysis were combined with standard Laemmli buffer and heated to 85°C to denature for 5 minutes. Protein samples were size separated on NuPAGE® 12% Bis-Tris gels (Invitrogen) in 2-(N-morpholino)ethanesulfonic acid (MES) SDS running buffer at 150 Volts. SeeBlue® Plus-2 prestained standard (Invitrogen) was used to visualize and estimate the progression and size of the sample migration. Gels were then transferred to 0.2 µm nitrocellulose using Towbin buffer (25 mM Tris, 192 mM glycine, pH 8.6) containing 20% methanol at 150 mA for 1 hour. For Aβ western blotting, membranes were incubated at 95°C for 1 minute in PBS to allow for improved antigen binding and then cooled in room temperature PBS prior to blocking. Membranes were blocked in 2% non-fat dry milk (NFDM) PBS for 1 hour. Between all remaining steps, membranes were washed 3× for 10 minutes each with PBS-T (0.05% Tween 20). For detection of APP, the mouse monoclonal 6E10 was used at 1 µg/mL in 2% NFDM PBS overnight at 4°C. Bound primary antibodies were detected using a sheep anti-mouse-HRP (#NA931V, GE Healthcare) at 50 ng/mL in 2% NFDM PBS and then developed with ECL Advance Reagent (GE Healthcare) followed by exposure to film emulsion.

### Immunohistochemistry

Staining was done on 50 µm free-floating sections, as described previously [15]. Primary antibodies were monoclonal HJ3.4 antibody (0.75 µg/ml), polyclonal rabbit anti panAβ (1 µg/ml), polyclonal rabbit anti APP (0.5 µg/ml), polyclonal sheep anti tau (1 µg/ml), polyclonal rabbit anti pS199 (1 µg/ml), and monoclonal PHF1 antibody (1∶1000). Corresponding biotinylated secondary antibodies and Vectastain Elite strepavidin-biotin kit (Vector Laboratories) were used for detection. 3-3′diaminobenzidine substrate was used for color development.

### Stereology

All stereological quantifications were done via StereoInvestigator version 8.2 software, as previously described [15]. Quantifications were done such that the injury status and survival time post injury were blinded to the experimenter. Optical fractionator stereological method was used to estimate number of APP-, HJ3.4-, and total tau-positive axonal bulbs in the ipsilateral fimbria and tau-positive somata in ipsilateral amygdala. The spherical probes (aka “space balls”) method was used to estimate tau-positive process length in the contralateral CA1 region. Parameters for counting grids and counting frames were as previously reported [15].

### Statistical methods

All data were analyzed using Prism 5.0 (GraphPad Sofware, Inc). For changes of either Aβ or tau pathology as function of time, one-way ANOVAs with Newman-Keuls post tests were used because there were no prespecified hypotheses about the direction of change. For pairwise comparisons of Aβ immunohistochemical data between injured 3xTg-AD and APP/PS1 mice, and of tau immunohistochemical data between 3xTg-AD and Tau_P301L_ mice, student's t-tests were employed. Values are expressed as means ± SEM. Statistical significance was set at p<0.05.
